# Minocycline-Induced Angioedema With Chronic Recurrent Urticaria Even After Drug Withdrawal: A Case Report

**DOI:** 10.7759/cureus.95786

**Published:** 2025-10-30

**Authors:** Kritikka Ajit Kumar, Saurabh Dubey

**Affiliations:** 1 Division of Translational Medicine, Department of Internal Medicine, University of South Florida Morsani College of Medicine, Tampa, USA; 2 Department of Internal Medicine, State University of New York Downstate Health Sciences University, Brooklyn, USA

**Keywords:** adverse drug event, anaphylaxis, angioedema, antibiotic allergy, chronic urticaria, dermatographism, drug hypersensitivity, minocycline reaction

## Abstract

Minocycline is a tetracycline antibiotic widely prescribed for several conditions, including acne, rosacea, and chlamydial and staphylococcal infections. Although cutaneous adverse reactions are known, severe immediate hypersensitivity requiring intensive care is rare. Even less common is prolonged urticaria persisting after drug discontinuation. A 16-year-old female patient developed diffuse urticaria and angioedema requiring intensive care within four days of minocycline initiation. Despite discontinuation and acute management, recurrent urticarial episodes occurred for over 12 months without re-exposure, requiring intermittent corticosteroid treatment. Causality assessment using the Naranjo Adverse Drug Reaction Probability Scale (score 7) indicated a probable association between minocycline and the observed reaction. This report highlights an uncommon clinical course of minocycline-induced angioedema with chronic recurrent urticaria persisting long after drug withdrawal.

## Introduction

Minocycline is a tetracycline antibiotic widely prescribed for chlamydial infections, skin and soft-tissue infections caused by* Staphylococcus aureus*, rosacea, and acne vulgaris [1]. Although cutaneous adverse reactions are well-documented, severe type I hypersensitivity requiring intensive care is uncommon [2]. Even rarer is the development of chronic urticaria after drug discontinuation.

Among tetracycline-associated adverse effects, drug reaction with eosinophilia and systemic symptoms (DRESS) represents a delayed hypersensitivity syndrome characterized by fever, morbilliform rash, lymphadenopathy, and internal organ involvement that typically develops within 2-6 weeks of drug exposure. A comprehensive review of antibacterial antibiotic-induced DRESS identified 254 cases, of which 21 (8.3%) were linked to tetracycline therapy [3]. While DRESS is most often associated with prolonged exposure, acute and immediate hypersensitivity reactions to minocycline are far less common and not as well recognized.

This report describes a 16-year-old female patient who developed diffuse urticaria and angioedema requiring intensive care within four days of initiating minocycline. Despite prompt discontinuation and standard treatment, she experienced recurrent urticarial episodes for over 12 months without re-exposure, necessitating intermittent corticosteroid therapy.

Causality assessment using the Naranjo Adverse Drug Reaction Probability Scale indicated a probable association [4]. This case underscores an unusual course of minocycline-induced angioedema with prolonged recurrent urticaria and highlights the need for clinician vigilance, appropriate risk stratification, and long-term monitoring of patients exhibiting hypersensitivity reactions to tetracyclines.

## Case presentation

A 16-year-old female patient with polycystic ovary syndrome (PCOS) and no known allergies was prescribed oral minocycline at a dosage of 200 mg daily for acne vulgaris. On day 4 of treatment, the patient developed diffuse pruritus, hives, and generalized edema. She was not taking any medications other than minocycline, nor did she report exposure to any new food or environmental agents. Minocycline was discontinued, and oral antihistamines were initiated for these symptoms, and she was sent back home. On day 5, however, her symptoms worsened despite oral antihistamine therapy, and the patient presented with fever, vomiting, hypotension, and symptomatic dermographism to the emergency department and subsequently to the intensive care unit (ICU) with a working diagnosis of anaphylaxis and angioedema, consistent with the World Allergy Organization guidelines on anaphylaxis [5,6]. Management included intramuscular epinephrine, intravenous corticosteroids, and antihistamines. The patient improved clinically and was discharged after eight days on a tapering course of oral steroids with advice to avoid tetracyclines. The rapid onset, absence of systemic organ involvement, and prompt steroid responsiveness were consistent with an acute IgE-mediated hypersensitivity reaction rather than a delayed eosinophilic or DRESS-type response, and so the above diagnosis of anaphylaxis and angioedema was made on clinical grounds. 

During follow-up, despite strict avoidance of minocycline and other tetracyclines, the patient experienced recurrent urticarial wheals predominantly on the palms and soles over a 12-month period. Several of these flares required short courses of oral corticosteroids, and symptoms resolved spontaneously after one year.

**Figure 1:**
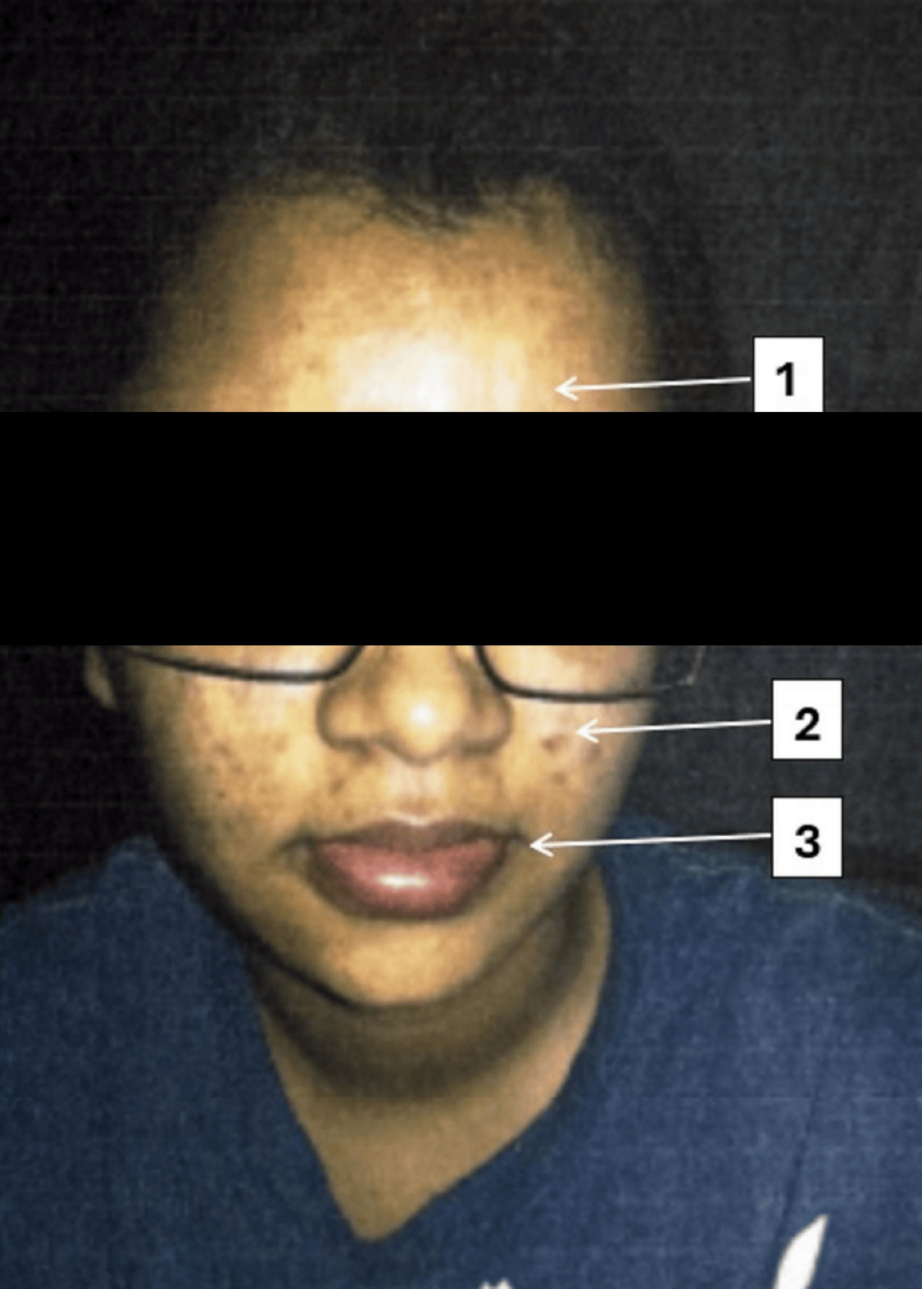
Clinical manifestations of allergic reactions with associated dermatological findings Clinical photograph of the patient demonstrating multiple dermatological features. (1) Facial edema presenting as diffuse swelling over the forehead. (2) Acne vulgaris with inflammatory lesions noted over the right cheek. (3) Angioedema involving the perioral region with swelling of the upper and lower lips

Alternative diagnoses considered included viral or bacterial exanthems (ruled out by spontaneous resolution without antimicrobial therapy), Stevens-Johnson syndrome (ruled out by the absence of mucocutaneous erosions), and leukemia cutis (excluded by rapid steroid responsiveness). Collectively, these findings supported a hypersensitivity etiology, and a final diagnosis of minocycline-induced angioedema with chronic recurrent urticaria was established (Figure 1, Table 1).

**Table 1:** Timeline of clinical events and management in a patient with minocycline-induced angioedema and recurrent urticaria This table summarizes the patient’s clinical course from the initiation of drug treatment through recovery

Timepoint	Clinical events	Management/outcome
Day 0	Started oral minocycline 200 mg daily	Apparently healthy
Day 4	Diffuse pruritus, hives, generalized edema	Minocycline stopped; oral antihistamines started
Day 5	Fever, hypotension, dermatographism	Epinephrine, IV steroids, ICU admission
Days 6–8	Gradual recovery	Steroid taper; discharge
Months 1–12	Recurrent urticaria without re-exposure	Short steroid bursts; resolution

## Discussion

Clinical context

The temporal relationship, clinical presentation, and improvement in acute symptoms after withdrawal strongly support minocycline as the causative drug in this case. Most reports of minocycline-induced urticaria or angioedema describe resolution shortly after drug withdrawal, which makes the persistence of symptoms in our patient unusual [1,2]. This emphasizes the importance of adhering to international urticaria management guidelines, which highlight the need for extended follow-up and stepwise escalation of therapy in patients with persistent disease [7,8].

Causality assessment

Causality assessment was done using the Naranjo Adverse Drug Reaction Probability Scale (Table 2). It yielded a score of 7, classifying the event as a probable adverse drug reaction (scores of 0 are classified as 'doubtful', 1-4 are classified as 'possible', 5-8 as 'probable', and 9 or more as 'definite'. Of note, certain items within the scale could not be scored because the patient was not challenged with minocycline again, given the severity of the drug reaction. This was supported by ICU records documenting the initial anaphylactic presentation and the absence of alternative etiologies.

**Table 2:** Naranjo Adverse Drug Reaction Probability Scale for minocycline in this patient Naranjo Adverse Drug Reaction Probability Scale, demonstrating a total score of 7, consistent with a probable adverse drug reaction to minocycline [6]

Question	Answer	Score
1. Are there previous conclusive reports on this reaction?	Yes	+1
2. Did the adverse event appear after the suspected drug was given?	Yes	+2
3. Did the adverse reaction improve when the drug was discontinued or a specific antagonist was given?	Yes	+1
4. Did the adverse reaction reappear when the drug was readministered?	Not done	0
5. Are there alternative causes that could have caused the reaction?	No	+2
6. Did the reaction reappear when a placebo was given?	Not applicable	0
7. Was the drug detected in blood (or other fluids) in toxic concentrations?	Not done	0
8. Was the reaction worsened with increased dose or lessened with decreased dose?	Not applicable	0
9. Did the patient have a similar reaction to the same or similar drugs in the past?	No	0
10. Was the adverse event confirmed by any objective evidence?	Yes	+1
Total Score		7

Possible mechanism

Angioedema is a rare but serious adverse effect of minocycline therapy and has been documented almost exclusively in female patients [1]. Chronic urticaria persisting beyond drug discontinuation suggests a sustained immune disturbance. Minocycline’s high tissue affinity may induce long-term immune dysregulation. Initial exposure likely triggered persistent immune activation and reduced regulatory T-cell function, sustaining mast-cell and basophil hyperreactivity despite drug avoidance.

A previously described case involved a patient with PCOS, a condition associated with autoimmune tendencies [9]. However, the limited number of reports prevents the establishment of a definitive correlation between PCOS and minocycline-related hypersensitivity. The observation remains speculative and warrants further investigation.

DRESS and differential diagnosis

DRESS should be considered in patients with fever, morbilliform rash, lymphadenopathy, leukocytosis with eosinophilia, and visceral organ involvement (commonly hepatic or renal). The condition typically manifests two to six weeks after exposure and occurs more often in females. In a review of 254 reported antibiotic-induced DRESS cases, tetracyclines accounted for 8.27 % (21 cases) [9]. The patient in this case had no concomitant medications, excluding confounding agents. Alternative diagnoses such as viral exanthem, Stevens-Johnson syndrome, and serum-sickness-like reactions were ruled out based on clinical course and recovery.

Management and therapeutic considerations

First-line therapy for chronic urticaria involves non-sedating oral antihistamines. If inadequate, addition of montelukast or low-dose doxepin may be considered. In persistent disease, systemic corticosteroids can control acute exacerbations but require cautious tapering due to adverse effects. Sulfasalazine may be employed as a steroid-sparing agent for long-term management. Refractory cases may require immunomodulators such as methotrexate or cyclosporine, although toxicity limits their use. Omalizumab, a recombinant anti-Immunoglobulin E monoclonal antibody, has shown substantial benefit in patients unresponsive to conventional therapy [10,11]. 

## Conclusions

This case demonstrates that minocycline can precipitate not only acute angioedema but also chronic urticaria that persists long after discontinuation of therapy. The prolonged course observed in our patient underscores the importance of recognizing minocycline as a potential trigger for autoimmune-mediated hypersensitivity, particularly in young female patients. Clinicians should maintain vigilance when prescribing tetracyclines, ensure close follow-up in cases of hypersensitivity, and consider long-term monitoring for patients who develop persistent or recurrent urticarial symptoms.
